# Effect of Music Intervention on Improving the Early Prognosis of the Preterm Infant in Chongqing, China: A Randomized Controlled Trial

**DOI:** 10.3390/children11121522

**Published:** 2024-12-16

**Authors:** Furong Shen, Lei Bao

**Affiliations:** 1 Department of Pediatrics, University-Town Hospital of Chongqing Medical University, Chongqing 401331, China; sfr@hospital.cqmu.edu.cn; 2 Department of Neonatology, Ministry of Education Key Laboratory of Child Development and Disorders, Key Laboratory of Pediatrics, Children’s Hospital of Chongqing Medical University, Chongqing 400014, China

**Keywords:** music intervention, music therapy, preterm infants, vital sign, weight gain

## Abstract

Objectives: To investigate the effects of music intervention on the vital signs, weight gain, feeding, hospital stays, and cost of premature infants. Methods: 100 premature infants were randomized into two groups: the experimental group (given music for 30 min at a time, once every day until discharge) and the control group (without music). To compare the vital signs (RR, HR, SPO_2_) before, during, and after the music intervention, as well as the weight gain and feeding, follow up to 3 months after discharge. Results: Although respiratory rate (RR) showed a decreasing trend at certain time points during and after music exposure, these changes did not reach statistical significance after adjusting for multiple comparisons. The experimental group had a shorter time to regain birth weight (6.07 ± 2.47 days) compared to the control group (8.93 ± 4.31 days) and a shorter time of intravenous nutrition (8.6 ± 3.87 days vs. 11.66 ± 5.85 days). The experimental group also exhibited a lower fasting rate, a faster-sucking speed, a lower hospital stay (10.36 ± 4.36 days vs. 12.46 ± 5.73 days), lower cost, higher NBNA scores, and a lower re-hospitalization rate within 3 months after the first discharge. Conclusions: Early music intervention may contribute to the growth and development of preterm infants, improve feeding, reduce hospitalization duration and costs, and improve short-term prognosis, though effects on respiratory rate require further study with a larger sample size.

## 1. Introduction

The current global annual incidence of premature births is approximately 13 million, representing over 10 percent of total live births [1]. Preterm birth and its complications currently constitute the primary cause of child mortality, accounting for over 20% of all deaths in children under the age of five. Survivors of preterm birth may encounter long-term health implications, including an elevated risk of disability and developmental delays [2]. China is a very populated country with over 10 million infants born each year, with the number approaching 10 million in 2022. The incidence of premature babies in China has also shown an increasing trend year by year, from 4% to 5% in the 1990s, gradually rising to the current 7% to 10% [3]. According to the annual report of The Chinese Neonatal Network (CHNN) [4], in 2020, preterm infants under 32 weeks of gestation had a survival percentage of 96.1% with comprehensive therapy, while preterm infants weighing less than 1500 g had a survival rate of 95.6%. Effective nursing interventions are essential for the early intervention of preterm newborns in order to optimize their prognosis and growth and development process. This study focuses on preterm infants with a gestational age of ≥32 weeks, as this population presents a relatively stable condition suitable for evaluating the effects of music intervention. Infants with a gestational age of <32 weeks are often placed in specialized units due to their need for invasive respiratory support and are therefore excluded from the current study. Additionally, the noise from mechanical ventilation in these units may interfere with the therapeutic effects of music.

Singing, playing, and listening to music are examples of planned music activities that are professionally adapted and supported by evidence in the case of premature infants through music therapy [5,6,7]. Its goal is to facilitate the bonding process between preterm newborns and their parents by attending to their varied sensory, weight, and emotional demands [8,9]. The study and application of neonatal music therapy in neonatal intensive care units (NICUs) is growing. The effects of music therapy on preterm infants have been extensively investigated in numerous studies [10,11,12,13,14]. Premature infant oral motor intervention [PIOMI] combined with music therapy was found effective for the feeding progression of premature infants, and infants who received both these interventions were discharged sooner than control infants [15]. In addition, music reduced fatigue in mothers who had preterm newborns in the NICU [16,17]. Some researchers also found that parent-led, infant-directed singing supported by a music therapist resulted in no significant differences between groups in mother–infant bonding, parental anxiety, or maternal depression at discharge [18]. Taking Chongqing of China as an example, this paper investigated the effects of music intervention on vital signs, weight gain, feeding, hospital stays, and the cost of premature infants.

## 2. Data and Method

### 2.1. Clinical Data

**Figure 1:**
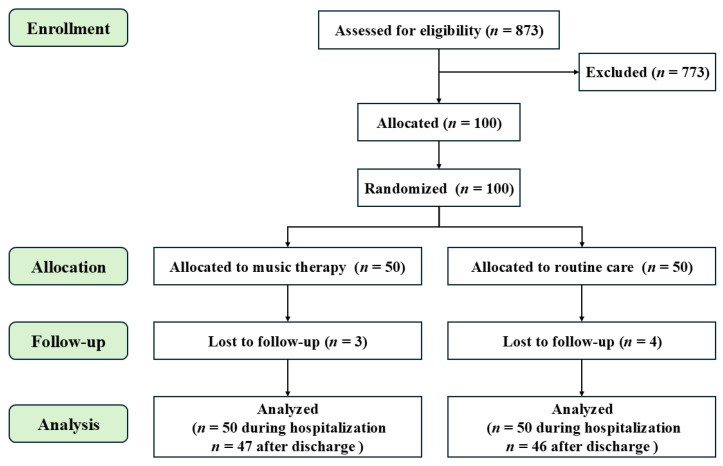
Research Flowchart.

Preterm infants hospitalized in our department were randomly divided into a control group and a music group, with 50 cases in each group, and the basic information is shown in Figure 1 and Table 1. Both groups were fed with the same kind of formula milk, and preterm infants in both groups were supplemented by intravenous nutrition when they had insufficient daily calorie requirements. The differences between the two groups in terms of sex, gestational age, chronological age, birth weight, Apgar score, underlying diseases, and feeding methods were not statistically significant and were comparable. Preterm infants with a gestational age of ≥32 weeks were selected for this study as they represent a relatively stable group appropriate for music therapy intervention. Infants under 32 weeks are routinely transferred to a separate unit in our department due to their critical condition and the requirement for invasive respiratory support. The noise from mechanical ventilation in these units may reduce the feasibility and effectiveness of the intervention, making them unsuitable for inclusion in the current study.

### 2.2. Trial Method

This study was a prospective randomized trial. Groups were determined by hospital bed number: the odd number was included in the experimental group, and the even numbers were included in the control group. Moreover, the hospital beds were allocated by other staff who were not involved in this research. The control group was given routine care every day, and the music group (trial group) was given music intervention based on routine care. Music was played in the incubator at 12:00 noon every day for 30 min once a day, starting from the 2nd to the 3rd day of hospitalization until discharge. We chose Mozart’s music, ‘Twinkle, Twinkle, Little Star,’ and several Chinese children’s songs. The music was played by a music player (XiaoMi^®^, Xiaomi Corporation, Beijing, China), which was placed at a distance of 20–25 cm from the infants’ heads in the incubator, and the volume monitored by a decibel detector (TASI TA8151, Suzhou TASi Electronics Co., Ltd., Suzhou, China) was controlled at 50–55 dB [19] while background noise was maintained below 45 dB. The lights were dimmed when the music was played, and the incubator was covered with a shading cloth to keep the surroundings quiet and to avoid invasive nursing operations. The observation was conducted from 10 min before the music started to 60 min after the music ended. If music was played antenatally, the same music was played; if the parents did not play music antenatally, then they chose Mozart’s music [20]. During the process of playing music, the preterm baby’s reaction was closely observed. If the baby cried, frowned, or shook his/her limbs repeatedly, the music was stopped immediately; the music was played again after 10 min. If a similar reaction occurs, the music will not be played again on the same day to avoid overstimulation. If similar reactions occurred on two consecutive days, the infant was excluded from the study. Inclusion and exclusion criteria are listed in Table 2. If the infant’s milk intake was below 20 mL per feeding or the total daily intake was less than 70–80 mL/kg/day, parenteral intravenous nutrition was administered. Once the infant’s milk intake reached 20 mL or more per feeding, or the total daily milk intake reached 80 mL/kg/day, a small amount of 5% glucose solution was supplemented, if necessary, to meet total fluid volume requirements.

### 2.3. Evaluation Index

The following indices were evaluated: vital signs monitoring (respiration, heart rate, and oxygen saturation recorded before, during, and after music sessions), weight gain assessment (daily body weight measurements, comparisons of average daily weight gain, weight loss during hospitalization, and days to regain birth weight between the two groups), feeding and sucking function analysis (comparisons of average daily milk intake, intravenous nutrition days, and fasting rate due to severe feeding intolerance (the fasting rate is defined as the ratio of the number of fasting cases during hospitalization to the total number of cases in the group), along with assessments of sucking speed and decrease in oxygen saturation during sucking both with and without music in the music group), hospitalization duration and cost, neurological function development evaluation (NBNA score at the appropriate age), and post-discharge follow-up (until 3 months after discharge to assess sleep, mood, and any re-hospitalizations).

### 2.4. Statistical Method

Randomized grouping method: Infants were allocated to groups based on their hospital bed numbers within a specific interval. Odd-numbered beds were assigned to the experimental group, while even-numbered beds were assigned to the control group. Bed allocation in the control group was uniformly managed by the full-time staff of the department, with no involvement from the researchers in the allocation process. SPSS 22.0 statistical software for data processing, the measurement information was expressed as MEAN ± S, and the data were analyzed by ANOVA, *T*-test, and chi-square test.

## 3. Results

### 3.1. Vital Signs

The third day of the music intervention was selected as the observation day. A 100 min observation period was recorded, starting 30 min after the completion of feeding to ensure that feeding-related effects on vital signs were minimized. Observations were made 10 min before the start of the music (baseline), 10, 20, and 30 min after the start of the music, and 30 min (60 min in Table 1) and 60 min (90 min in Table 1) after the end of the music. Respiratory rate, heart rate, and oxygen saturation were measured at each time point and compared with the baseline. The results are presented as follows:

#### 3.1.1. Respiratory Rate

The respiratory rate (RR) at each observation point is shown in Table 3. Initial ANOVA analysis indicated apparent differences in RR at 20 min after the start of the music, 30 min after the start of the music, and 30 min after the end of the music when compared with the baseline. However, after applying the Bonferroni correction to account for multiple comparisons, with an adjusted significance level of 0.01, these differences were no longer statistically significant. Table 3 reflects the *p*-values after the Bonferroni correction, indicating no statistically significant differences after multiple comparison adjustments.

#### 3.1.2. Heart Rate

The heart rate (HR) at each observation point is shown in Table 4. ANOVA analysis indicated no statistically significant differences between each observation point and the baseline before or after the Bonferroni correction. Thus, no statistically significant changes in heart rate were observed across the observation period. Therefore, we report no statistically significant changes in heart rate throughout the observation period.

#### 3.1.3. Oxygen Saturation

The oxygen saturation (SpO_2_) at each observation point is shown in Table 5. ANOVA analysis showed no statistically significant differences between each observation point and the baseline before or after Bonferroni correction, confirming no significant changes in oxygen saturation across time points. Thus, we report no statistically significant changes in oxygen saturation throughout the observation period.

### 3.2. Weight Gain

The average daily weight gain and weight loss during hospitalization and the number of days to regain birth weight in the two groups are shown in Table 6. No statistically significant differences were found between the two groups for average daily weight gain and weight loss during hospitalization. Although the difference in days to regain birth weight was observed, this difference did not reach statistical significance after multiple comparison corrections.

### 3.3. Feeding

#### 3.3.1. Two Groups: Average Daily Milk Intake

The average daily milk intake of the music group was 137.99 ± 40.97 mL/day, and the average daily milk intake of the control group was 128.01 ± 48.85 mL/day. The difference between the two groups was not statistically significant (*p* = 0.271), and the results are shown in Table 7.

#### 3.3.2. Two Groups: Time of Application of Intravenous Nutrition

A comparison of the duration of application of intravenous nutrition during hospitalization between the two groups is shown in Table 6. The duration of intravenous nutrition in the music group was 8.60 ± 3.87 days, and the duration of intravenous nutrition in the control group was 11.66 ± 5.85 days. The difference between the two groups was statistically significant (*p* < 0.05).

#### 3.3.3. Two Groups: Fasting Rate and Average Fasting Time During Hospitalization

In the music group, 8 infants fasted during hospitalization (fasting rate of 16%), the longest fasting time was 174 h, the shortest fasting time was 8 h, and the average fasting time was 84.94 ± 58.92 h; in the control group, 19 infants fasted (fasting rate of 38%), the longest fasting time was 138 h, the shortest fasting time was 5 h, and the average fasting time was 63.16 ± 41.68 h (χ^2^ = 6.139, *p* = 0.013).

#### 3.3.4. Music Group: Sucking Speed with/Without Music

The 4th day of music intervention was designated as the observation day, during which the feeding was assessed at two different time points. The interval between these observations exceeded 6 h, with one time point involving music intervention and the other serving as a control. A comparison was made on the same day to determine the time required for 50 premature infants in the music group to consume an equivalent volume of milk with and without music. Additionally, their sucking speed was calculated and presented in Table 8. In the music group, feeding occurred at a rate of 16.03 ± 14.38 mL/min with music intervention compared to 11.01 ± 6.75 mL/min without it, demonstrating a statistically significant difference (*p* < 0.05).

#### 3.3.5. Music Group: SPO_2_ Decrease When Feeding with/Without Music

The oxygen saturation levels during feeding were recorded for both preterm infants in the music group and those without music. The lowest oxygen saturation value observed during the sucking process was documented, and the difference between the oxygen saturation before feeding and this minimum level represented a measure of decreasing oxygen saturation. The findings are presented in Table 8. Oxygen saturation decreased by (5.08 ± 2.26)% when accompanied by music compared to (6.30 ± 2.35)% without music, indicating a statistically significant difference (*p* < 0.05). While this difference is statistically significant, the clinical relevance of a 1.22% difference in oxygen saturation decrease is relatively limited, as it falls within the range of expected physiological variability in preterm infants. However, this finding highlights the potential of music intervention to stabilize oxygen saturation during feeding, which could be beneficial for infants prone to feeding-related desaturation.

### 3.4. Hospitalization Days and Cost

The comparison of hospitalization days and hospitalization costs between the music group and the control group is as follows (Table 9). The difference between the two groups was statistically significant (*p* < 0.05).

### 3.5. Follow-Up After Discharge

Further follow-up, 3 months after discharge, 47 patients in the music group completed follow-up, of whom 3 were lost to follow-up, and 46 patients in the control group completed follow-up, of whom 4 were lost to follow-up. The two groups were followed up as follows:

#### 3.5.1. NBNA Score Comparison

The neurobehavioral development was assessed using the NBNA score in both the music intervention group and the control group at 42 weeks of corrected gestational age. In the music intervention group, a total of 35 participants completed the NBNA score, while in the control group, 31 participants completed it. The average NBNA score for the music intervention group was 36.94 ± 1.59 points, whereas for the control group, it was 36.13 ± 1.69, indicating a statistically significant difference between these two groups (*p* = 0.048).

#### 3.5.2. Sleep and Mood Status

A total of 47 parents in the music group completed the follow-up, of whom 44 (93.6%) were satisfied that their children slept better after discharge from the hospital, did not cry frequently, and were easily calmed. In the control group, 46 parents completed the follow-up, of whom 39 (84.8%) had the above situation.

#### 3.5.3. Re-Hospitalization 3 Months After Discharge

During the follow-up period until 3 months after discharge, 6 patients in the music group were re-hospitalized for diseases, including 4 cases of pneumonia, 1 case of rotavirus enteritis, and 1 case of gastroenteritis. In the control group, 11 were readmitted to the hospital, including 8 cases of pneumonia, 1 case of diarrhea, 1 case of jaundice, and 1 case of infectious hydrocele.

## 4. Discussion

### 4.1. Effect of Music Intervention on the Vital Signs of Preterm Infant

Premature infants often exhibit shallow, rapid, and irregular breathing due to their immature respiratory systems. Compared with full-term infants, preterm infants have fewer alveoli, leading to reduced gas exchange and an inability to maintain stable oxygen saturation. Additionally, the cardiovascular system function in preterm infants is often unstable. Previous studies have demonstrated that music can improve cardiopulmonary function in premature infants by reducing respiratory rate (RR) and heart rate and enhancing oxygen saturation [21,22,23,24,25,26].

In our study, we observed a gradual decrease in respiratory rate after the initiation of music. Initially, statistical analysis indicated significant differences in RR at 20 min and 30 min after the start of the music and at 30 min after the end of the music when compared to baseline. However, after applying the Bonferroni correction to account for multiple comparisons, these differences were no longer statistically significant. This suggests that while there may be a trend toward reduced RR with music intervention, the observed effect did not reach statistical significance after multiple comparison corrections, potentially due to sample size limitations. The lack of statistically significant findings after correction could be attributed to the conservative nature of the Bonferroni adjustment combined with the limited sample size, which may have reduced the power to detect small but potentially meaningful changes in RR.

Heart rate and oxygen saturation also showed some degree of change during the music intervention, though these changes were not statistically significant, both before and after correction. This lack of significant findings could be related to our relatively small sample size and the fact that most infants were in stable condition with minimal fluctuations in vital signs. The lack of significant changes in heart rate and oxygen saturation may also be attributed to the stable condition of most infants in this study, limiting the observable impact of music intervention. Future studies with larger sample sizes and the inclusion of infants with more severe conditions, such as neonatal respiratory distress syndrome (NRDS), may yield more significant results and provide further insights into the potential benefits of music intervention.

### 4.2. Effect of Music Intervention on the Weight Gain of Preterm Infants

Premature babies are born with low birth weight, and after birth, due to fluid loss, low milk intake, and other factors, weight loss can occur. This study found that the time for premature infants in the music group to recover their birth weight was shorter, which was consistent with the results of [27]. However, there was little difference in the average daily weight gain and weight loss between the two groups during hospitalization, which was inconsistent with the results of Kemper [28]. It may be related to short hospitalization days and insufficient observation time.

### 4.3. Effect of Music Intervention on the Feeding of Preterm Infants

Preterm infants have poor sucking ability and have not established perfect sucking, swallowing, and breathing function. The cardia relaxation, stomach volume is small, and vomiting can easily occur. Some digestive enzymes in the intestine are deficient, and they are more likely to suffer from feeding intolerance than full-term infants [29]. Studies have shown that music can make premature infants suck more forcefully, with faster frequency and duration, establish a complete sucking and swallowing function faster [30], and increase the total milk intake [31,32]. Although this study did not find that the average daily milk intake was higher in the music group, the time of intravenous nutrition was significantly lower than that in the control group, and there were fewer severe intolerances and fasting during hospitalization, faster sucking speed, and higher oxygen saturation was maintained during sucking. This study found that music intervention can improve the feeding of premature infants.

### 4.4. Effect of Music Intervention on the Hospitalization Days and Cost of Preterm Infant

Premature infants need to spend a period in the neonatal unit due to low weight, weak sucking ability, poor feeding tolerance, low immunity, and diseases. Music therapy has been shown to enhance the functionality of various organs and potentially reduce the duration of hospitalization [31,32]. This study found that music intervention can significantly shorten the average length of hospitalization and hospitalization costs of premature infants.

### 4.5. Effect of Music Intervention on the Sleep and Mood of Preterm Infants

This study was followed up to 3 months after the discharge of preterm infants and found that the music group of preterm infants slept better after discharge, cried less, and were more soothing, and parents were more satisfied. This is consistent with the study of [33]. At the same time, other studies have found that music can not only soothe premature babies but also relieve the anxiety of premature mothers and promote milk secretion [34].

### 4.6. Effect of Music Intervention on the Re-Hospitalization of Preterm Infants

In this study, 6 (12.77%) of the 47 preterm infants in the music group were hospitalized again within 3 months after discharge, while 11 (23.91%) of the 46 preterm infants in the control group were hospitalized again, and even some were hospitalized twice within 3 months after discharge, and the re-hospitalization rate was lower in the music group. Hamm [35] believes that this may be attributed to the improved recovery of organ functions and a more stabilized state observed in preterm infants in the music intervention group upon their discharge from the hospital.

### 4.7. Limitations of the Study

This study has several limitations. First, while statistically significant differences were observed in physiological parameters such as oxygen saturation and feeding volume, the clinical relevance of these differences may be limited due to the short observation period. Second, the inclusion criteria focused on infants with a gestational age ≥32 weeks and a birth weight > 1500 g, excluding smaller and more immature infants who might benefit differently from music intervention. Future studies should consider including infants with a gestational age < 32 weeks or birth weight < 1500 g, as longer and more targeted music interventions might yield different or enhanced outcomes.

Third, the post-discharge assessment relied on the NBNA total score, which lacked detailed subscores. More comprehensive and standardized tools, such as TIMP or Bayley scales, with detailed evaluations of individual domains, could provide a clearer understanding of neurodevelopmental impacts. Additionally, post-discharge follow-up on sleep and behavioral outcomes was based on qualitative reports rather than quantifiable measures. Employing standardized tools, such as validated questionnaires, would allow for a more precise and objective analysis.

Finally, the absence of a power calculation may affect the robustness of the study’s conclusions. Addressing these limitations in future research through larger sample sizes, long-term follow-up, and detailed, quantifiable outcome measures would provide stronger evidence for the clinical relevance of music intervention in preterm infants.

## 5. Conclusions

In conclusion, music therapy, as a non-invasive intervention, can significantly improve the feeding of preterm infants with relatively stable conditions, promote growth and development, shorten the length of hospitalization, reduce hospitalization costs, and reduce the rate of re-hospitalization. Music therapy is anticipated to offer a novel approach to enhancing the health, prognosis, and overall quality of life for premature infants, thus optimizing its benefits for this vulnerable population.

## Figures and Tables

**Table 1:** Comparison of basic information between the music group and control group.

Characteristic		Music Group	Control Group	*p*
sex	male	28	22	0.230
	female	22	28	
gestational age (weeks)		34.6 ± 1.44	34.4 ± 1.27	0.404
chronological age (days)		1.80 ± 1.31	1.42 ± 1.34	0.155
birth weight (g)		2122.8 ± 212.2	2090.2 ± 201.2	0.432
Apgar scores	1′	8.80 ± 1.03	8.86 ± 0.762	0.762
	5′	9.7 ± 0.51	9.46 ± 0.079	0.079
	10′	9.92 ± 0.27	9.84 ± 0.223	0.223
Underlying disease/case	Neonatal pneumonia	42	40	0.680
	Neonatal sepsis	3	4	
	Neonatal hyperbilirubinemia	4	5	
	Neonatal hypoglycemia	1	0	
	Congenital heart disease	0	1	

**Table 2:** Inclusion and exclusion criteria.

Criteria	Details
Inclusion	gestational age: 32 weeks–36 + 6 weeks
	birth weight: 1500–2500 g
	tolerating part of enteral nutrition ^1^
	without invasive mechanical ventilation
	having passed a hearing screening test
Exclusion	the hearing screening (TEOAE) failed on both sides
	severe brain damage ^2^ and congenital malformations in critical condition ^3^
	states considered unsafe ^4^ by medical specialists
	termination requested by their parents

Note: 1. In this study, there was no specific quantitative standard (e.g., mL/kg/day) for tolerating partial enteral nutrition. Infants were included as long as they could tolerate oral feeding, including minimal feeding. 2. Refers to severe brain injuries such as asphyxia requiring hypothermia therapy, Grade III or higher intraventricular hemorrhage, etc. 3. Examples include esophageal atresia, congenital diaphragmatic hernia, or severe congenital heart diseases such as tetralogy of Fallot. 4. Refers to conditions where the infant’s status is unstable, such as requiring mechanical ventilation or hemodynamic instability requiring vasopressors.

**Table 3:** Respiratory rate before, during, and after music intervention (inspiration/min, MEAN ± S).

Observation Point	−10 min Baseline	10 min	20 min	30 min	60 min	90 min
RR	48.74 ± 7.44	45.98 ± 7.41	45.78 ± 6.86	45.66 ± 6.25	45.26 ± 6.23	46.58 ± 6.31
*p*	/	0.054	0.039	0.032	0.015	0.131

Note: After Bonferroni correction for multiple comparisons (adjusted significance level = 0.01), none of the time points maintained statistical significance, indicating that the observed trend may not represent a robust effect and could be influenced by sample size limitations.

**Table 4:** Heart rate before, during, and after music intervention (beat/min, MEAN ± S).

Observation Point	−10 min Baseline	10 min	20 min	30 min	60 min	90 min
HR	137.72 ± 10.49	137.72 ± 10.60	136.22 ± 9.29	136.42 ± 7.52	136.68 ± 8.08	137.14 ± 8.20
*p*	/	1.000	0.427	0.492	0.582	0.759

Note: As all *p*-values exceed the significance level of 0.05, Bonferroni correction does not affect the conclusion. No statistically significant differences were observed in heart rate across time points.

**Table 5:** Oxygen saturation before, during, and after music intervention (%, MEAN ± S).

Observation Point	−10 min Baseline	10 min	20 min	30 min	60 min	90 min
SPO_2_	96.52 ± 1.39	96.7 ± 1.69	96.96 ± 2.04	96.94 ± 1.88	96.78 ± 1.75	96.68 ± 1.79
*p*	/	0.615	0.219	0.241	0.467	0.655

Note: All *p*-values exceed the significance level of 0.05; hence, the Bonferroni correction does not affect the conclusion. No statistically significant differences in oxygen saturation were observed across time points.

**Table 6:** Comparison of weight during hospital stay between the music group and control group (MEAN ± S).

	Average Daily Weight Gain (g)	Weight Loss (g)	Days to Regain Birth Weight (days)
music group	12.71 ± 15.1	75 ± 59.22	6.07 ± 2.47
control group	10.65 ± 13.30	96.2 ± 57.59	8.93 ± 4.31
*p*	0.47	0.073	0.000

**Table 7:** Comparison of average daily milk intake and intravenous nutrition time between the music group and the control group.

Group	Average Daily Milk Intake (mL/day)	Intravenous Nutrition Time (day)
music group	138.00 ± 40.97	8.56 ± 3.87
control group	128.01 ± 48.85	11.66 ± 5.85
t	1.107	−3.087
*p*	0.271	0.003

**Table 8:** Comparison of sucking speed and decrease in oxygen saturation of the music group with and without music.

Group	Sucking Speed (mL/min)	Decrease in Oxygen Saturation (%)
music group	16.03 ± 14.38	5.08 ± 2.26
control group	11.01 ± 6.75	6.3 ± 2.35
t	3.106	3.025
*p*	0.003	0.004

**Table 9:** Comparison of hospital stay and expense between music group and control group.

Group	Hospital Stay (day)	Hospital Expense (¥)
music group	10.36 ± 4.36	16,404.33 ± 5910.74
control group	12.46 ± 5.73	21,259.98 ± 14,692.90
t value	−2.062	−2.168
*p*	0.042	0.034

## Data Availability

The dataset generated for this study will be made available by the corresponding author on request in an anonymized version to guarantee a low risk of patient identification.
